# Supplementary material for: Development of a novel PIK3CA-mutated pancreatic tumor mouse model and evaluation of the therapeutic effects of a PI3K inhibitor
Source: PLoS One. 2025 Jul 10;20(7):e0326491. doi: 10.1371/journal.pone.0326491 (PMC12244556; doi:10.1371/journal.pone.0326491)
Supplement: S1 Fig — DNA was extracted from KPC/PPC mouse tails or KPC/PPC cells and underwent Polymerase Chain Reaction (PCR). Lanes 1 and 2 indicated that the LSL-PIK3CAH1047R sequence was included in PPC mice and PPC cells. Lanes 3 and 4 indicated that recombination of LSL-PIK3CAH1047R sequence occurs only in PPC cells. Lane 5–8 indicated that KPC mice and cells did not have LSL-PIK3CAH1047R sequence. The primer sequences are as follows:>PIK3CA: detecting the sequence of PIK3CAH1047R/+ Forward: 5’-gtgtgccagagcaagtcattg −3’ Backward: 5’-atgacggcatggtgaagctat −3’ >re-PIK3CA: detecting recombination of LSL-PIK3CAH1047R/+Forward: 5’- ggttgaggacaaactcttcgc −3’ Backward: 5’- ttcgtcttgcagaagctgatg −3’ (PDF) [file pone.0326491.s001.pdf]

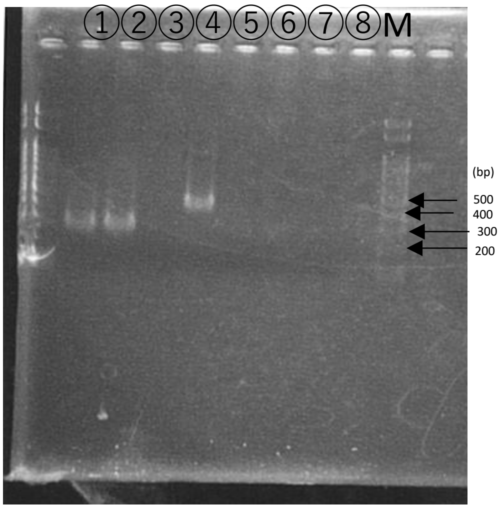

Lane	sample	primer
1	PPC mice tail	PIK3CA
2	PPC cells	PIK3CA
3	PPC mice tail	re-PIk3CA
4	PPC cells	re-PIk3CA
5	KPC mice tail	PIK3CA
6	KPC cells	PIK3CA
7	KPC mice tail	re-PIk3CA
8	KPC cells	re-PIk3CA
M	markers	

S1 Fig. The image of nucleic acid electrophoresis of PPC/KPC mice and cells on an agarose gel.

DNA was extracted from KPC/PPC mouse tails or KPC/PPC cells and underwent Polymerase Chain Reaction(PCR).

Lanes 1 and 2 indicated that the LSL-PIK3CA^{H1047R} sequence was included in PPC mice and PPC cells.
Lanes 3 and 4 indicated that recombination of LSL-PIK3CA^{H1047R} sequence occurs only in PPC cells.
Lane 5-8 indicated that KPC mice and cells did not have LSL-PIK3CA^{H1047R} sequence.

The primer sequences are as follows:
>PIK3CA: detecting the sequence of PIK3CA $^{H1047R/+}$
Forward: 5’-gtgtgccagagcaagtcattg -3’
Backward: 5’-atgacggcatggtgaagctat -3’
>re-PIK3CA: detecting recombination of LSL-PIK3CA $^{H1047R/+}$
Forward: 5’- ggttgaggacaaactcttcgc -3’
Backward: 5’- ttcgtcttgcagaagctgatg -3’
