# Supplementary material for: Development of a novel PIK3CA-mutated pancreatic tumor mouse model and evaluation of the therapeutic effects of a PI3K inhibitor
Source: PLoS One. 2025 Jul 10;20(7):e0326491. doi: 10.1371/journal.pone.0326491 (PMC12244556; doi:10.1371/journal.pone.0326491)
Supplement: S2 Fig — The area in red frame is used in Fig (A)2A, (B)2B and (C)4A. Molecular size (kDa) are indicated on the right side of the membrane. (B) These blots are cut off before hybridization with the antibody. (PDF) [file pone.0326491.s002.pdf]

(A)

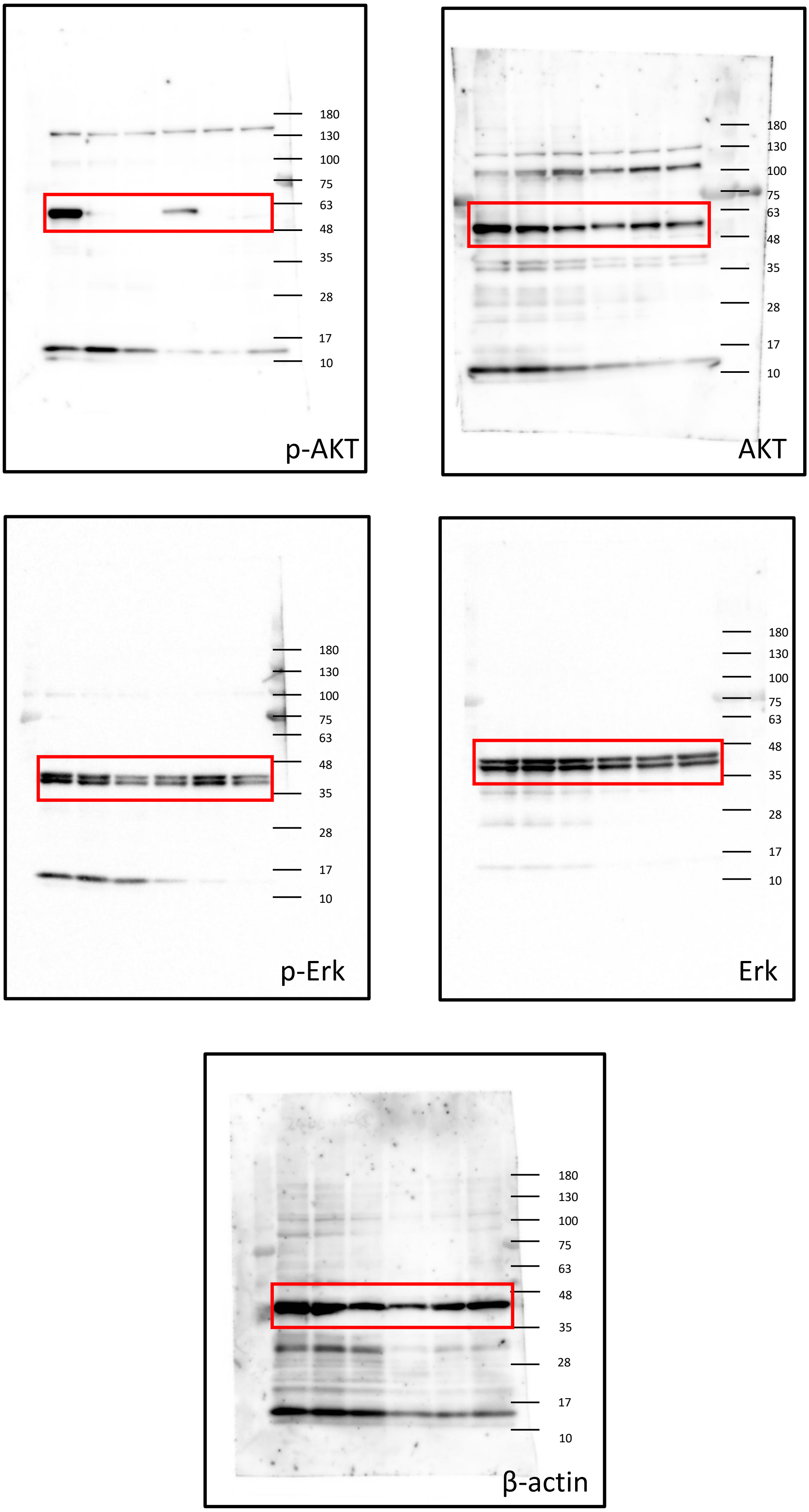

S2 (A) Fig. Full blots of Western blotting assay in Figure 2A.

The area in red frame is used in Figure 2A.
Molecular size (kDa) are indicated on the right side of the membrane.

(B)

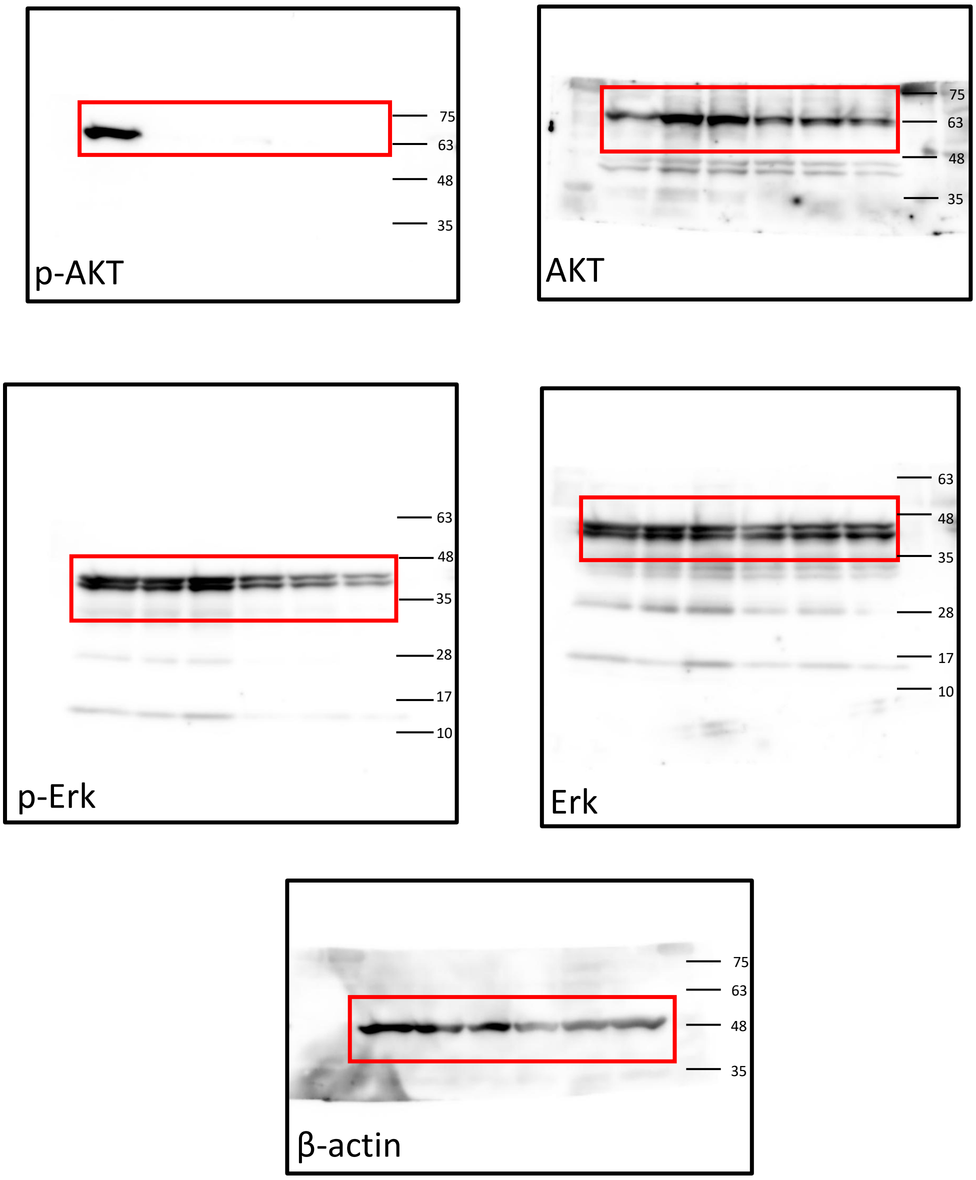

S2 (B) Fig. Full images of Western blotting assay in Figure 2B.

The area in red frame is used in Figure 2B.
Molecular size (kDa) are indicated on the right side of the membrane.
These blots are cut off before hybridization with the antibody.

(C)

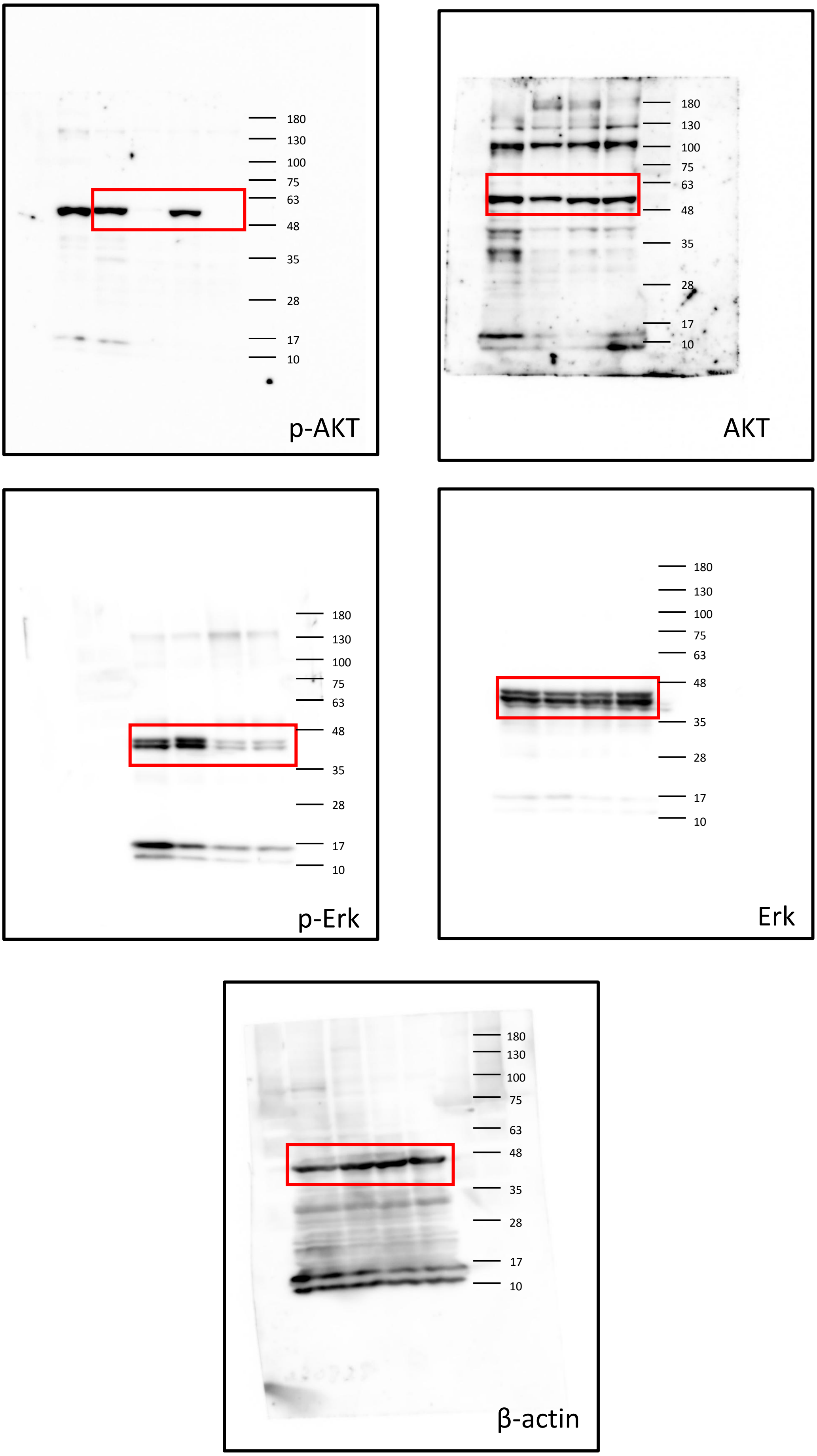

S2 (C) Fig. Full blots of Western blotting assay in Figure 4A.

The area in red frame is used in Figure 4A.
Molecular size (kDa) are indicated on the right side of the membrane.
