# Supplementary material for: Development of a novel PIK3CA-mutated pancreatic tumor mouse model and evaluation of the therapeutic effects of a PI3K inhibitor
Source: PLoS One. 2025 Jul 10;20(7):e0326491. doi: 10.1371/journal.pone.0326491 (PMC12244556; doi:10.1371/journal.pone.0326491)
Supplement: S1 Table — The sex, tumor weight, body weight, histology, and metastasis of PC, PPC, and KPC mice. KPC mice (Ptf1aER–Cre/+ KrasG12D/+ p53loxP/loxP) was fed tamoxifen at 6 weeks of age and dissected at 18 weeks of age. (PDF) [file pone.0326491.s003.pdf]

> PPC mice(day150)

No.	sex	BW*(g)	PW** (g)	PW/BW (%)	Histology	metastasis
PPC-1	m	32.2	0.73	2.27	PDAC†	No
PPC-2	m	32.3	0.40	1.24	PanIN†† with Cysts	No
PPC-3	m	22.2	0.61	2.75	PanIN with Cysts	No
PPC-4	m	32	0.42	1.31	PanIN	No
PPC-5	m	35.3	0.77	2.18	PanIN	No
PPC-6	m	33.6	0.50	1.49	PanIN	No
PPC-7	f	24.8	0.38	1.53	PanIN	No
PPC-8	f	25.9	0.67	2.59	PDAC with cysts	No
PPC-9	f	24.6	0.20	0.81	PanIN with Cysts	No
PPC-10	f	25.6	0.95	3.71	PDAC with cysts	No

> PC mice(day250)

No.	sex	BW(g)	PW (g)	PW/BW (%)	Histology	metastasis
PC-1	m	35.1	0.85	2.42	PanIN	No
PC-2	m	39.2	0.78	1.99	No tumor	No
PC-3	m	32	0.34	1.06	PanIN with cysts	No
PC-4	m	31.1	0.49	1.58	PanIN with cysts	No
PC-5	f	32.6	0.28	0.86	PanIN with cysts	No
PC-6	f	25.8	0.45	1.74	PDAC with cysts	No
PC-7	f	31.3	0.49	1.57	PanIN with cysts	No
PC-8	f	28.1	0.46	1.64	ADM	No

> PPC mice(day100)

No.	sex	BW(g)	PW (g)	PW/BW (%)	Histology	metastasis
PPC-11	m	22.1	0.32	1.45	PanIN with cysts	No
PPC-12	m	23.6	0.36	1.53	PanIN	No
PPC-13	m	28.8	0.39	1.35	ADM†††	No

> PC mice(day150)

No.	sex	BW(g)	PW (g)	PW/BW (%)	Histology	metastasis
PC-9	m	25.1	0.34	1.35	No tumor	No
PC-10	m	28.9	0.44	1.52	small ADM	No
PC-11	f	23.2	0.44	1.90	small ADM	No

>PPC mice(day50)

No.	sex	BW(g)	PW (g)	PW/BW (%)	Histology	metastasis
PPC-14	m	25.5	0.32	1.25	No tumor	No
PPC-15	m	24.3	0.40	1.65	Small ADM	No
PPC-16	m	24.4	0.30	1.23	Small ADM	No

> KPC mice

No.	sex	BW(g)	PW (g)	PW/BW (%)	Histology	metastasis
KPC-1	m	29.8	0.2	0.67	High Grade PanIN	No
KPC-2	m	24.9	0.7	2.81	PDAC	No
KPC-3	m	28.1	0.46	1.64	PDAC	No
KPC-4	m	22.6	0.27	1.19	High Grade PanIN	No
KPC-5	f	21	0.44	2.10	PDAC	No
KPC-6	f	22.1	0.28	1.27	High Grade PanIN	No
KPC-7	f	22.4	0.17	0.76	High Grade PanIN	No
KPC-8	f	20	0.77	3.85	PDAC	No

*BW; Body weight **PW; Pancreas weight

†PDAC; Pancreatic ducal adenocarcinoma

††PanIN; Pancreatic intraductal neoplasia

†††ADM; Acinar Ductal Metaplasia

S1 Table. The details of PC, PPC, and KPC mice

The sex, tumor weight, body weight, histology, and metastasis of PC, PPC, and KPC mice.
KPC mice (Ptf1a$^{ER\text{–}Cre/+}$ Kras$^{G12D/+}$ p53$^{loxP/loxP}$) was fed tamoxifen at 6 weeks of age and dissected at 18 weeks of age.
