# Supplementary material for: Development of a novel PIK3CA-mutated pancreatic tumor mouse model and evaluation of the therapeutic effects of a PI3K inhibitor
Source: PLoS One. 2025 Jul 10;20(7):e0326491. doi: 10.1371/journal.pone.0326491 (PMC12244556; doi:10.1371/journal.pone.0326491)
Supplement: Attachment — Submitted filename: Point by point Response.docx [file pone.0326491.s005.docx]

Point-by-point response to reviewers

Dear reviewers,

We would like to thank the editors and reviewers for reviewing our manuscript and their valuable comments.

**Response to Reviewer #1**

*>1. Stock numbers for each mouse strain should be included in methods.*

We included them in methods.

*>2. Cat# for all reagents, drugs, including antibodies should be included in methods.*

We included them in methods.

*>3. Provide more details of in vivo study, how many mice per gender, per timepoint were evaluated for tumors.*

We provided the details of *in vivo* study in supplementary information, table S1. PPC mice were evaluated on day 50 (three males), day 100 (three males), and day 150 (six males and four females). PC mice were on day150 (two males and one female) and day250 (four males and four females). KPC mice were at 18weeks of age, four males and four females.

*>4. include pancreas weights data for each mouse evaluated, all strains and all time points.*

We included pancreas weight data in table S1.

*>5. Compare the pancreas weights and lesion development between PC, PPC and KPC strains. Was there any difference between genders?*

Histological findings for each mouse are described in table S1. PC and PPC mice mainly developed tumors with cysts, but no cystic lesions were found in KPC mice. There were no gender difference in PPC mice (day150), PC mice

*>6. Was PC strain also evaluated at 50 and 100 days or just at 250 day? if yes what was the finding? if no ...why?*

PC strain was evaluated at 150 and 250 day. At 150 day, almost no tumors had developed in the pancreas.

*>7. Is alpelisib able to inhibit mutant PIK3CA? discuss and provide reference.*

According to the previous report (PMID: 24608574), Alpelisib (BYL719) also inhibits PIK3CA H1047R mutation. We inserted the following sentence in the fourth paragraph in the Background section and included the article in the references; “this inhibitor also inhibits PIK3CA E545K and H1047R mutation.”

*>8. Figs are not clear, need high resolution images for reviewing.*

We apologize for the inconvenience. Please see the attached PDF file. You can see the figures more clearly.

*>9. Why did they use subcutaneous model, instead of transgenic model itself for evaluating drug effect.*

In transgenic model, because it’s difficult to measure tumor size without dissecting the mice, the changes of tumor size over time cannot be measured. However, we can easily measure the size in subcutaneous model. So, subcutaneous model is adequate for the evaluation of the effects of alpelisib although the transgenic model more closely resembles human pancreatic tumors.

**Response to Reviewer #2**

*>1. With regard to the precision of the manuscript, it is essential to cite relevant references for the facts and methods presented in the manuscript accurately. The first four comments address this requirement. Please cite the correct reference regarding the statement: “While PIK3CA mutations are relatively common in breast, gastric, and colorectal cancers”. It is not mentioned in the 10th reference.*

We include the reference (PMID: 20535651).

*>2. Please cite references regarding the following sentence: Several mutation hotspots have been recognized in PIK3CA, and gain-of-function mutations such as E545K and H1047R are critical to carcinogenesis.*

We include the reference (PMID: 16322248).

*>3. Please cite references regarding the following sentence: Thus, the indications of these PI3K/AKT inhibitors in pancreatic cancer are limited. However, for pancreatic cancer, which currently has few molecular therapy targets, the potential application of these PI3K/AKT inhibitors is needed to improve prognosis.*

We include the references (PMID: 34503244, 28754816)

*>4. Please cite the reference for the mentioned guideline: “Reporting of In Vivo Experiments guidelines.”*

We include the reference (PMID: 32663219).

*>5. I recommend substituting 'development' for 'generation' in the titles, as ‘development’ is more appropriate for the context.*

We changed ‘generation’ to ‘development’ in the title.

*>6. Since you evaluated different effects of alpelisib, I recommend substituting 'effects' for 'effect' in the titles.*

We changed ‘effect’ to ‘effects’ in the title.

*>7. Please substitute the terms 'mouse' or 'mice' with 'murine' in the manuscript. This change enhances precision, as 'murine' can also refer to rats.*

We changed ‘murine’ to ‘mouse’ in the manuscripts.

*>8. This part of the manuscript is related to the mice part of the materials and methods section: “While considering the humane endpoint, it was ensured that the tumors did not reach a size of 10 mm in any dimension. Mice were observed once every 3 days for the humane endpoints mentioned above. The duration of the experiments was about 250 days after birth. A total of 20 mice was used and euthanized. None of the mice was found dead during the experiments. Animal health and behavior were monitored every 3 days. All considerations for welfare were taken to minimize suffering and distress due to the creation of tumors. All animals were humanely euthanized via gradual CO2 exposure followed by cervical dislocation.”*

We moved this part to the ‘mice’ part.

*>9. Ethical statements should be included at the end of the Materials and Methods section after the Statistical Analysis section.*

We moved ‘Ethical statements’ to the end of the Method section.

*>10. Please specify the sleep-wake cycle established for the mice.*

We included the following sentence in the mice section of Method: “The mice were housed under a 12-hour light/12-hour dark cycle, with lights on at 7:00 AM and off at 7:00 PM.”

*>11. Please define fetal bovine serum (FBS) when it is first mentioned.*

We defined fetal bovine serum(FBS) in the “cell culture”section in Method.

*12. Please provide a justification for the dosage and timing of alpelisib administration.*

In the present study, alpelisib was administered at 40mg/kg for two weeks (5 out of 7 days). In the referenced study #28 (PMID: 30051890), it was administered at 45mg/kg for 15days. our dosage and timing of alpelisib administration is not exactly the same, but reasonable.

*>13. Please specify the limitations of your study before stating the conclusion.*

We added the following limitation before conclusion; “This study has several limitations. First, more than 90% of human pancreatic cancers are Kras-positive [13], but PPC mice do not exhibit Kras mutation. The phenotypes of mice with both Kras and PIK3CA mutations remain unclear, including their drug sensitivity and responses to PI3K and Kras inhibitors, and warrant further investigation. Second, the effect of alpelisib for PPC tumor was evaluated in a subcutaneous tumor model. Although the transgenic model more closely resembles human pancreatic tumors, it is difficult to measure tumor size over time. Therefore, our results may not reflect the effect in human pancreatic tumors due to differences in the tumor microenvironment. Finally, we have not performed in vivo experiments with the combination of alpelisib and a MEK inhibitor.”

*>14. Please include the supporting information files in which the data are available.*

We provided supporting information in which the data are available.

**Respose to Reviewer #3***:*

*>The PI3K/AKT pathway plays a crucial role in the progression of pancreatic cancer. However, there is no precedent for the successful use of PI3K inhibitors in the treatment of pancreatic cancer. Researchers have developed a new mouse pancreatic cancer model with the PIK3CA H1047R mutation. They established a pancreatic cancer cell line from PPC mice, and the PI3K p110α inhibitor alpelisib can inhibit the proliferation of PPC cells in vitro. This model is of great value for the subsequent search for therapeutic drugs for PI3K/AKT - driven pancreatic cancer.*

*>This study lacks direct evidence to verify the PIK3CA H1047R mutation. It is recommended that the authors provide information on the detection of PIK3CA mutations in cell lines using PCR.*

We provided PCR picture in supplementary figure S1.

We included the following sentence in “Effect of alpelisib on PPC and KPC cells *in vitro*” section in RESULT; “Recombination of the PIK3CA gene in PPC cells was confirmed by Polymerase Chain Reaction (Figure S1).”
