# Supplementary material for: Development of a novel PIK3CA-mutated pancreatic tumor mouse model and evaluation of the therapeutic effects of a PI3K inhibitor
Source: PLoS One. 2025 Jul 10;20(7):e0326491. doi: 10.1371/journal.pone.0326491 (PMC12244556; doi:10.1371/journal.pone.0326491)
Supplement: Attachment — Submitted filename: Point by point Response2.docx [file pone.0326491.s006.docx]

Point-by-point response to editors and reviewers

Dear editors and reviewers,

We would like to thank the editors and reviewers for reviewing our manuscript and their valuable comments.

**Response to editors**

*>1. Firstly, please mention correct journal information in the cover/rebuttal letter.*

We sincerely apologize for the error in the journal name mentioned in our cover letter.

We have corrected it and submitted the revised version accordingly.

*>2. Secondly, in the first page of the manuscript there is a : 'These authors contributed equally to this work', but there was no respective indicator related to that.*

We have now added the appropriate asterisks and daggers to the author names on the title page to indicate equal contributions, in accordance with the journal's guidelines.

**Response to Reviewer #2**

*>1. Compared to the referenced study (PMID: 30051890), the administration of alpelisib was approximately 69% lower. Please provide a rationale for this reduction.*

Thank you for your valuable comment.

The dose of alpelisib used in preclinical studies varies across reports. A previous study (PMID: 32899250) demonstrated that a dose of 15 mg/kg administered over four weeks was sufficient to achieve therapeutic efficacy. The total drug exposure in that study is comparable to that in our current study, supporting the validity of the dosing regimen we employed. Therefore, we believe our selected dose is appropriate and justified.

We have cited this reference in the Methods section under the description of the subcutaneous tumor allograft model.
