# Supplementary material for: Development of a novel PIK3CA-mutated pancreatic tumor mouse model and evaluation of the therapeutic effects of a PI3K inhibitor
Source: PLoS One. 2025 Jul 10;20(7):e0326491. doi: 10.1371/journal.pone.0326491 (PMC12244556; doi:10.1371/journal.pone.0326491)
Supplement: Attachment — Submitted filename: Point by point Response 3.docx [file pone.0326491.s007.docx]

Point-by-point response to editors

Dear editors,

We would like to thank the editors for reviewing our manuscript and their valuable comments.

**Response to editors**

*>Please follow PLOS guidelines properly to indicate corresponding author and multiple groups of equally contributed authors.*

We sincerely apologize for the incorrect statement regarding the contributing authors.

The author contributions have been accurately entered in the submission form, and we have now removed the equal contribution note from the manuscript.
